# A Practical Simulation Method to Calculate Sample Size of Group Sequential Trials for Time-to-Event Data under Exponential and Weibull Distribution

**DOI:** 10.1371/journal.pone.0044013

**Published:** 2012-09-05

**Authors:** Zhiwei Jiang, Ling Wang, Chanjuan Li, Jielai Xia, Hongxia Jia

**Affiliations:** 1 Department of Health Statistics and the Ministry of Education Key Lab of Hazard Assessment and Control in Special Operational Environment, School of Preventative Medicine, Fourth Military Medical University, Xi'an, Shaanxi, China; 2 Department of Endocrinology, Jinan General Hospital, Jinan, Shandong, China; National Institute of Environmental and Health Sciences, United States of America

## Abstract

Group sequential design has been widely applied in clinical trials in the past few decades. The sample size estimation is a vital concern of sponsors and investigators. Especially in the survival group sequential trials, it is a thorny question because of its ambiguous distributional form, censored data and different definition of information time. A practical and easy-to-use simulation-based method is proposed for multi-stage two-arm survival group sequential design in the article and its SAS program is available. Besides the exponential distribution, which is usually assumed for survival data, the Weibull distribution is considered here. The incorporation of the probability of discontinuation in the simulation leads to the more accurate estimate. The assessment indexes calculated in the simulation are helpful to the determination of number and timing of the interim analysis. The use of the method in the survival group sequential trials is illustrated and the effects of the varied shape parameter on the sample size under the Weibull distribution are explored by employing an example. According to the simulation results, a method to estimate the shape parameter of the Weibull distribution is proposed based on the median survival time of the test drug and the hazard ratio, which are prespecified by the investigators and other participants. 10+ simulations are recommended to achieve the robust estimate of the sample size. Furthermore, the method is still applicable in adaptive design if the strategy of sample size scheme determination is adopted when designing or the minor modifications on the program are made.

## Introduction

In the past few decades, group sequential design has been widely used in clinical trials. The sample size calculation is of particular importance when designing a group sequential trial. Adequate sample size guarantees the power to detect the difference and too large sample size is undesired due to the considerations of study time and costs. Various methods, such as Pocock design, O'Brien-Fleming design, the error-spending function, *etc*, were proposed to design the group sequential trials [1]–[3] and their approaches of sample size estimation for continuous and dichotomous variables were also discussed [4]–[5]. Compared with normal and binary data, sample size estimation for time-to-event data is more complicated because of its ambiguous distributional form and censored data. However, the time-to-event outcomes are frequently employed in clinical trials. For example, the study endpoints, such as overall survival (OS), progression free survival (PFS) and time to progression (TTP), are usually adopted as primary endpoints in oncology clinical trials. The survival analysis is necessary to analyze the time-to-event data. The group sequential design is often indispensible to oncology trials for ethical considerations. Therefore, it will be helpful to have a practical and simple method to calculate the sample size when group sequential design is applied in clinical trials with time-to-event endpoint.

For survival data, the exponential and Weibull distribution are the two most frequently used parametric models. Of the two distributional forms, the Weibull distribution is more appropriate to describe time-to-event data than the exponential distribution in most cases because it includes the shape parameter besides the scale parameter, which is also contained in the exponential distribution. The shape parameter in the Weibull distribution makes it possible to describe the varied hazard rate over the study interval, which is common in medical studies. Recently, several approaches were proposed to estimate/re-estimate the sample size for group sequential and adaptive design [6]–[13]. But few of them were considered under the assumption of the Weibull distribution. Only Murphy *et al* [6], Togo *et al* [7] and Lu *et al* [9] discussed the effects of varied shape parameters under the Weibull distribution on the sample size in two-stage clinical trials. Most of these methods were introduced under the assumption of the exponential distribution [8], [10]–[13]. The Weibull distribution is seldom employed in the commercial tools, too. For example, PASS is only able to calculate the sample size for survival data under the exponential distribution [14]. Though Heo *et al* proposed a formula to calculate the sample size for survival data under the Weibull distribution [15], it is just for traditional single-stage trials and cannot be applied in multi-stage group sequential design. Furthermore, the proposed approaches shown in these literatures are usually based on the particular statistical tests or trial procedure. They are sometimes complicated in the actual clinical trials. On the other hand, the Monte Carlo simulation-based approach is an easy and flexible method to estimate the sample size for a trial which has a particular trial design, trial procedure, statistical test and target power [16]. The changes of the trial procedure and other demands on the trial can be conveniently incorporated into the simulation so that the accurate sample size is calculated. The simulation-based method is also generally adopted in some commercial softwares, such as, PASS, nQuery, *etc*. But the Weibull distribution, which is more suitable to describe the survival data, is not usually considered in these softwares. Therefore, a practical and simple method, which employs Monte Carlo simulation to calculate the sample size for multi-stage two-arm group sequential trials with time-to-event endpoint, is proposed in this paper and its SAS program has been developed. Here, both the exponential and Weibull distribution are considered and the simulation is based on the log-rank test. The probability of discontinuation in the trial is also included and leads to a more accurate estimate. Besides the recommended sample size, a series of assessment indexes will be calculated in the program to determine the optimal plan of group sequential trial. To call the program for sample size determination, a method of estimating the shape parameter based on the hazard ratio and median survival time is suggested from the practical perspective when the Weibull distribution is assumed. Moreover, the generalization of the method for adaptive design is discussed.

## Methods

### Parametric distributions of time-to-event data

Because various physical causes result in the occurrence of a specified event and it is impossible to isolate the causes and account mathematically for all of them, the theoretical distribution of time-to-event outcome is difficult to define in clinical trials [17]. It brings difficulties to sample size determination and statistical analysis of survival data. The common parametric distributions of survival data include exponential, Weibull, gamma, log-normal, log-logistic, normal, exponential power, Gompertz, inverse Gaussian, Pareto, generalized gamma distribution, *etc* [17]. Of them, the exponential and Weibull distribution are the two most important distributional forms in modeling the survival data and frequently employed to build up the survival parametric models.

The exponential distribution is the simplest and most important parametric distribution of time-to-event data. It is often referred to as a purely random failure pattern. The exponential distribution was firstly proposed to model failure data by Davis [18] and discussed why it had been selected to describe the survival data over the popular normal distribution by Epstein and Sobel [19]–[20]. Until now, it still plays a major role in modeling the time-to-event data due to its simplicity. The exponential distribution is characterized by a constant hazard rate 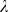, which indicates high risk and short survival. Let *t* be independent continuous time variable. For the survival data following the exponential distribution, the hazard rate at time *t* is defined as
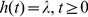
(1)and the survival rate at time *t* is
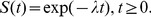
(2)Here, 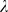 denotes the scale parameter of the exponential distribution. However, the constant hazard rate in the exponential distribution leads to the property of “lack of memory”, which appears too restrictive when employing it to describe the time-to-event data in clinical trials.

Unlike the exponential distribution, the varied hazard rate is considered under the Weibull distribution by including the shape parameter 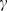 besides the scale parameter 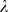. The distribution and its applicability were introduced to failure data by Weibull [21]–[22]. It is thought to be more appropriate to model the time-to-event data than the exponential distribution because of its varied hazard rate. The survival time following the Weibull distribution, has the hazard function
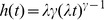
(3)and the survival function
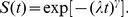
(4)Here, *t* is the independent continuous time variable. 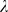 and 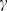 denote the scale and shape parameter of the Weibull distribution respectively. It is seen that the hazard rate increases when 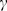
>1 and decreases when 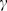
<1 as *t* increases. The exponential distribution is the special case of the Weibull distribution when 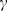 = 1 and the hazard rate is a constant.

Compared with other survival parametric distributions, the exponential and Weibull distribution are of particular importance and generally applied in modeling the time-to-event data in clinical trials. Although the simple form of the exponential distribution makes it easy to fit parametric models for survival outcomes, the Weibull distribution has its superiority, e.g., varied hazard rate, to describe the time-to-event data in medical studies. Therefore, both the exponential and Weibull distribution are employed to estimate the sample size of group sequential design for the time-to-event endpoint in the article.

### Group sequential design of time-to-event data

Compared with the traditional trial designs, group sequential design allows early stopping for efficacy/futility based on the results of interim analyses, which effectively improves trial efficiency, shortens trial duration and saves trial costs in some occasions. As a result, it was paid much attention from all trial participants and has been widely applied in clinical trials. When designing a group sequential trial, it is essential and important for biostatisticians to choose the optimal number of trial stages, interim time schedule and stopping boundaries of interim analyses [23]. To preserve the type I error probability in the group sequential design, many methods, e.g. Pocock design, O'Brien-Fleming design, the error spending function, [1]–[3] *etc*, were introduced to calculate the boundaries of early stopping for efficacy. The Pocock method averages the probability of early stopping in each interim analysis, while the O'Brien-Fleming method takes a conservative attitude at the early interim time points. It inflates the traditional single-stage sample size so little that it becomes one of the most widely used methods in group sequential design [24]. The error spending approach regards the trial as a process of consuming type I error rate. An increasing function 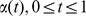, which characterizes the rate at which the type I error rate is spent, has to be prespecified in a particular trial and several functions were proposed by Lan *et al* [3]. Here, *t* denotes the fraction of the total information no matter whether information time or calendar time is employed in the trial [25]. The method is equivalent to Pocock design when 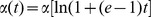 and O'Brien-Fleming design when 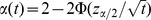. Depending on these methods, it is possible to perform extensive simulations to determine the optimal interim monitoring plan, including the number of trial stages and the time schedule of interim analyses, and calculate the sample size.

Although the methods we have mentioned above equally fit the survival trials, there are still some differences between the trials with survival and other types of endpoints. The definition of information time in the survival group sequential trials is different from the trials with normal or binary endpoint. The subjects provide full information to the survival trial only when the events occur. The information time at an interim analysis here is referred to as the proportion of maximum events already observed, but not all subjects who complete the trial [25]. Consequently, the sample size estimation means the calculation of the necessary number of the events for the subsequent stages in the survival trials [26]. Though the number of events in a trial guarantees the test power, the investigators and sponsors are more interested in the subjects to be accrued in a trial for the practical considerations. The transformation from the calculation of the number of events to the number of subjects is essential and vital for the sample size estimation of survival trials. Furthermore, the censoring in survival data makes it more complicated. Therefore, a Monte Carlo simulation-based method is employed and its SAS tool has been developed in this article. In the simulation, all these factors are incorporated to estimate the sample size. Both the number of necessary events to be observed and the number of subjects to be accrued are estimated. Moreover, besides the total sample size, a series of assessment indexes are calculated to help determine the optimal interim monitoring plan.

### The simulation-based method for sample size estimation

Here, we consider a two-arm group sequential trial with survival outcome. The hypothesis to be tested is
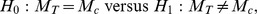
where 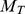 and 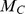 denote the median survival time of the treatment and control groups respectively. Under the assumption of proportional hazard, the hypothesis is equivalent to 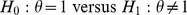, where 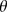 is the hazard ratio. Assuming that the trial is divided into *k* stages, the *i*-th (*i* = 1,2,…,*k*-1) interim analysis is performed after the *i*-th stage of the trial and the final analysis is implemented when all patients complete the trial. At the *i*-th interim analysis, the trial stops earlier for efficacy if the derived 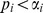, where 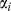 denotes the nominal significance level of the *i*-th stage. Or else, the trial continues to the subsequent stage. Early stopping for futility is not considered in this article. At the final analysis, the trial is considered to be successful when 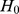 is rejected and failed when 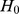 is accepted.

To calculate the sample size of survival group sequential trial, the trial parameters, which are related to the trial size, have to be prespecified besides the overall type I error rate 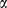 and the overall test power 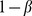. First of all, the median survival time 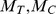 and the hazard ratio 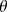 directly contribute to the size of the trial. Here, the hazard ratio can be derived by 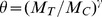 under the assumption of proportional hazard, where 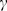 is the shape parameter of the Weibull distribution. A higher hazard ratio leads to a smaller sample size and a lower ratio brings a larger one when 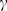 is fixed. 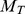 and 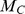 determine the scale parameters of the treatment and control groups respectively under the prespecified survival distribution. Secondly, the maximum observed time of the subject *T* is related to the sample size. The longer the patients are followed up in a trial, the smaller the sample size is needed. Thirdly, the interim monitoring plan, including the number of trial stages *k*, the interim time schedule 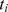 and the nominal significance level 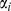 of interim analysis, *etc*, results in the trial size. Compared with the traditional single-stage design, multiple interim analyses in the group sequential design lead to sample size inflation with the given type I error rate and test power. The more stages are planned in a trial, the larger the sample size is needed. The degree of sample size inflation also depends on the nominal significance levels of interim analyses. As we have mentioned above, the O'Brien-Fleming boundaries result in the smallest sample size among the available approaches. The nominal significance levels can also be determined by performing extensive simulations as long as the overall type I error rate is well controlled. In addition, the survival distributional form is another important trial parameter for sample size estimation. Here, both the exponential and Weibull distribution are considered. It has to be prespecified to calculate the sample size in the simulation. When the Weibull distribution is assumed, the magnitude of shape parameter has to be defined at the same time. The drop-out rate and sample size ratio of the two groups are also necessary to calculate the sample size. In the simulation, it is presumed that the subjects be enrolled in sequence and the subject would enter the study only when the former one had finished the trial. The subject is considered to finish the trial if he/she dies, discontinues or has been followed up for the prespecified longest duration. Due to the presumption, only the subjects, who finish the trial at the interim analysis, are included for data analysis and no one is still at risk in the cohort at that time. Therefore, the accrual information, including the accrual time, accrual rate and accrual distribution, does not have the effects on the sample size. It is unnecessary to define them in the simulation.

In addition to the number of events to be observed and the number of subjects to be accrued in a trial, a series of assessment indexes are calculated to help determine the optimal interim monitoring plan in the simulation. They are listed and explained as follows.

(i) The stage-wise empirical power 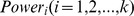 and cumulative empirical power 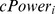 at the *i*-th stage. The stage-wise empirical power at the *i*-th stage is defined as the proportion of the simulative trials which reject the null hypothesis at the *i*-th stage when 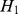 is true and calculated by 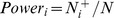, where 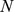 denotes the number of all simulative trials and 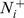 is the number of the ones which succeed at the *i*-th interim analysis. It reflects the probability of early stopping for efficacy at the *i*-th stage. The cumulative empirical power is calculated by 
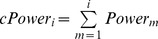
 and it equals to the overall empirical power when *i* = *k*. It should be noted that 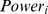 is just the stage-wise empirical type I error rate at the *i*-th stage when 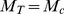 is assumed. Because it is referred to as the proportion of the trials which reject the null hypothesis at the *i*-th stage when 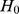 is true. Equally, 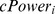 becomes cumulative empirical type I error rate under the null hypothesis. Therefore, as long as the various 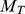 and 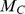 are prespecified, both the empirical power and type I error rate are calculated by employing the same program.

(ii) The expected number of events to be observed in a trial. For a *k*-stage group sequential trial with time-to-event outcome, let 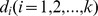 be the number of events to be observed at the *i*-th stage. The expected number of events to be observed in a trial is calculated by
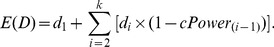
(5)Compared with the total number of events 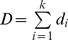, 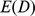 takes account of both the number of events to be observed and the probability of early stopping for efficacy. The cost-effective consideration is included to assess and determine the optimal interim monitoring plan.

**Figure 1:**
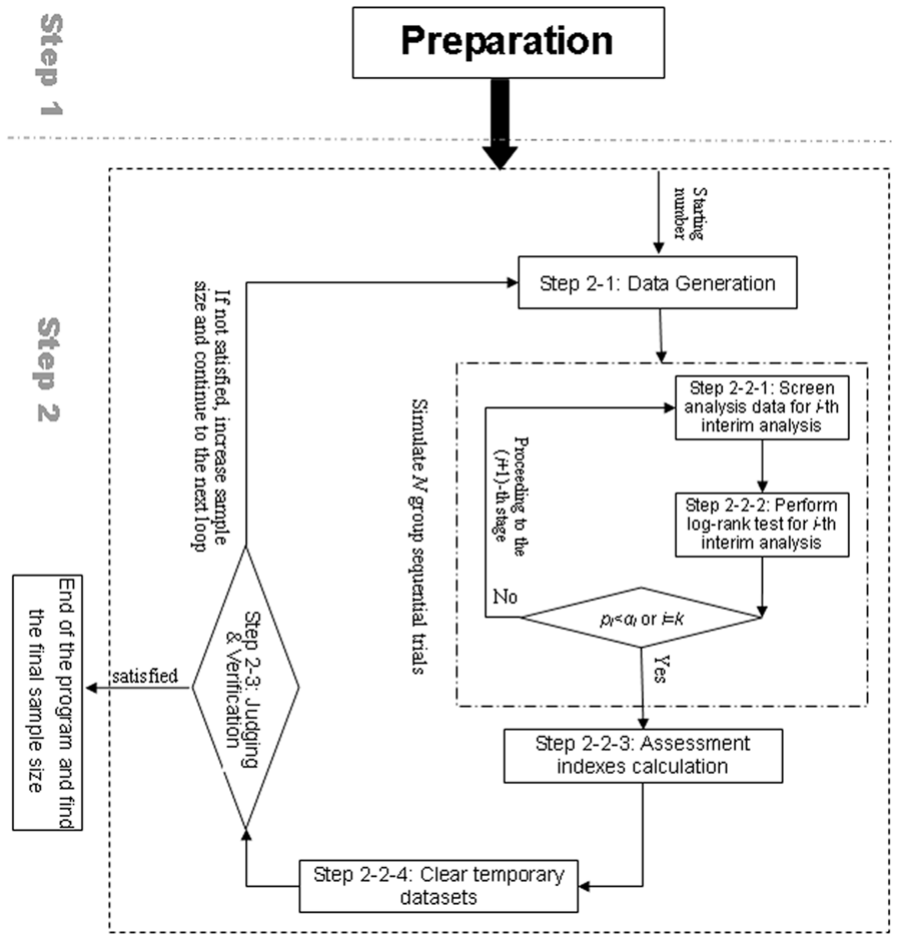
Program flow of the SAS macro.

Depending on the prespecified trial parameters, the Monte Carlo simulation based on the trial procedure is implemented to calculate the sample size, the number of events to be observed and other assessment indexes. In the simulation, the log-rank test is employed as the survival analysis method at the interim analyses and final analysis. All the subjects who finish the trial before the *i*-th stage are pooled for the *i*-th interim analysis. Correspondingly, a SAS macro %n_gssur based on the simulation-based method was developed to calculate the sample size and assessment indexes for survival group sequential design. The input macro parameters of the SAS macro are listed in Table 1. The run of the macro depends on the two sub-macros %exp_gen and %weibull_gen, in which the survival time is generated at random under the exponential and Weibull distribution respectively. The detailed program flow is designed according to the trial procedure and program requirements. It is descried in detail as follows and the flow chart is shown in Figure 1.

**Table 1:** The input macro parameters in the SAS macro %n_gssur.

Parameter	Definition
m1	The median survival time of the control group.
m2	The median survival time of the treatment group.
t	The maximum observed time of the trial.
dtr0	The survival distribution employed for sample size estimation. The options include ‘exp’ for exponential distribution and ‘weibull’ for Weibull distribution.
look	The number of stages in the trial.
info	The time points and their corresponding stopping boundaries for efficacy of the interim analysis.
min	The starting number of sample size searching of the control group.
max	The ending number of sample size searching of the control group.
len	The length of increment of sample size searching.
r	The sample size ratio of treatment and control groups.
drop	The drop-out rate of the trial.
power	The target overall power of the trial.
seed	The number used to generate a stream of reproducible random numbers.
sim	The number of simulated trials *N*.
path	The path and name to save the result text file.

#### Step 1

Prepare for the Monte Carlo simulation. The necessary trial parameters for sample size estimation are obtained from the macro parameters, which are shown in Table 1. The maximum follow up time, the sample size ratio, the dropout rate and the target power are defined according to the comments of clinical investigators and sponsors, which are descried in the trial protocol. For the information of interim monitoring plan, including the number, timing and boundaries of interim analyses, a series of scenarios are advised to try and the optimal plan is chosen by comparing the assessment indexes we have mentioned above. It will be illustrated in detail in the Results by using an example. The distributional form of the time-to-event data is defined according to the statistician's assumption and the shape parameter has to be given when the Weibull distribution is assumed, which is discussed detailedly in the Results and Discussion. The median survival time of the treatment and control group are prespecified based on the literature review and the expectation on the efficacy of the test drug. The scale parameter 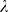 of the survival distribution and the survival rate of treatment and control groups at time *T* are calculated from the median survival time by employing Eq.(2) or Eq.(4) according to the assumed survival distribution. The other macro parameters are designed for the requirements of the program. Generally speaking, at least 1000 replications in the simulation are necessary for the robust estimate. The starting and ending number of the sample size searching can be defined according to the sample size of the traditional single stage design and the multiple attempts are indispensible.

#### Step 2

A series of loops are performed to search for the minimum sample size of the control group from the prespecified starting number until the target power or ending number is achieved. Let 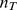 and 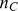 be the sample size of treatment and control groups respectively. The total sample size can be derived by 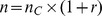, where *r* denotes the sample size ratio of 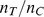. Given 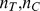, the drop-out rate 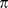 and the survival rate 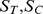 of the treatment and control groups at time *T*, the total number of preplanned events in the trial can be calculated by
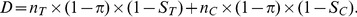
(6)Here, the drop-out rates of the two groups are assumed to be equal. Depending on Eq.(6), the number of events, which should be observed to achieve the target power, is derived when the final sample size is found in the loops. In each loop, the simulative trial with the particular sample size is generated randomly according to the prespecified survival distribution and analyzed based on the trial procedure. *N* trials are simulated to calculate the empirical power and other assessment indexes. The detailed procedures are described in sequence as follows.

#### Step 2-1

Generate the random survival time 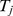 under the prespecified survival distribution. Here, the equal shape parameters across the two groups are assumed when the Weibull distribution is considered. When the data are generated, 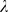 is given 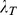 or 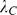 based on the treatment assignment of the subject, where 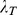 and 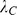 denote the scale parameter of the treatment and control groups respectively. The subject, who discontinues in the trial, is identified at random under the distribution of Bernoulli (*π*), where *π* denotes the drop-out rate of the trial and has to be prespecified in the macro parameter. Here, the subject is assumed to discontinue at random completely and the drop-out rates are equal across the two groups. The censored time of the discontinued subject is not cut down because it is assumed that they would not leave the trial earlier until the event is happening and they should be still from the same survival distribution as the one who finishes the trial.

#### Step 2-2

Simulate the procedure of group sequential design, perform the interim/final analysis and calculate the assessment indexes, e.g., the overall empirical power, the stage-wise empirical power, the expected number of events, *etc*. They are completed by the sub-steps as follows.

#### Step 2-2-1

Screen the subjects for interim analyses. When 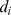 events are observed in the trial, the *i*-th interim analysis will be performed. The subjects who finish the trial before the *i*-th stage are screened for data analysis at the *i*-th interim analysis if no positive result is derived at the (*i*-1)-th interim analysis. Due to the conservative presumption that the subject would enter the study only when the former one had finished it, no subject is at risk at the interim analysis in the simulation.

#### Step 2-2-2

Perform the log-rank tests at the interim analyses and final analysis. If the derived 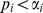 at the *i*-th interim analysis, the trial stops earlier for efficacy. Otherwise, it proceeds to the subsequent stage. If no positive result is concluded until the final analysis, the null hypothesis is accepted.

#### Step 2-2-3

Calculate the overall empirical power, 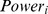, 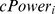 and the expected number of events to be observed for the particular sample size.

#### Step 2-2-4

Clear the temporary datasets at the end of the loop.

#### Step 2-3

Judge whether the target power is achieved and the minimum sample size is found. If the target power is achieved, the loops stop earlier and the final sample size is recommended. Otherwise, the loops continue until the prespecified ending number and the sample size searching fails. However, due to the fluctuation of the calculated empirical power for varied trial size in the simulation, the simulation will enter the stage of verification to check the stability of the calculated power when the target power is achieved for the first time. The trial size is denoted by 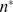 and considered as the initial estimate of the sample size of the control group. If the calculated empirical power is still larger than the target power for the trial size of 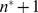 at the stage of verification, 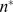 is considered as the recommended final sample size of the control group. Otherwise, the sample size of the control group will continue to increase by 1 until the target power is achieved for the second time. The trial size at that time is regarded as the recommended final sample size.

Moreover, censoring in the time-to-event data is another important issue to sample size estimation. Here, two types of right censored data are considered. One type of censored data consists of the subjects who discontinue in the trial. They are identified at random by employing Bernoulli distribution based on the prespecified drop-out rate. For simplicity, it is assumed that the drop-out subject would not leave the trial earlier until the event is happening and his/her censored time should be still from the same survival distribution as the one who finishes the trial. That is to say the survival time of the discontinued subject is also generated at the Step 2-1 in the simulation. They are only identified as the censored ones and the survival time does not change for discontinuation. Furthermore, the subjects, whose random survival time from the given survival distribution is longer than the maximum observed time, are considered to be censored because the events of these subjects cannot be observed at time *T*. The survival time of the subject is replaced by 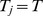.

## Results

A practical clinical trial is employed as an example to illustrate the use of the program and discuss the related issues. The primary objective of a placebo-controlled clinical trial was to assess the efficacy and safety of a new drug as the third-line treatment in the patients with metastatic colorectal cancer. The expected event of the trial was the death of the patient. The patient would be observed for 18 months at maximum until he/she died of the cancer. The primary endpoint of the trial was the OS of the patient. The sample size ratio of treatment and placebo groups was 2∶1 for ethical consideration. The group sequential design was considered by the investigators for the interests of the patients and early stopping for efficacy was anticipated. By literature review, the median OS of the placebo group was assumed to be 4.5 months. It was expected by the investigators that the test drug be able to lengthen the median OS by 1.5 months compared with the placebo. The sample size was calculated with the type I error rate of 0.05 and test power of 80%. The drop-out rate was given as 20%. The information time was employed for group sequential design and the O'Brien-Fleming boundaries were implemented to control the overall type I error rate. The SAS macro %n_gssur was employed to determine the optimal interim monitoring plan. The total sample size and the number of deaths to be observed were calculated at the same time. The decision process for the optimal plan was explained under the assumption of the exponential distribution for simplicity. The changes of total sample size and other assessment indexes for varied shape parameters under the Weibull distribution were also compared.

### (1) The decision process for the optimal interim monitoring plan

To choose the optimal interim monitoring plan, a series of scenarios were assumed to evaluate their differences and the interests of the patients. The scenarios included (A) traditional single stage trial, (B) two-stage trial with equal space time, (C) three-stage trial with equal space time and (D) three-stage trial with 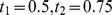. For practical consideration, the trial with ≥4 stages was not considered here. The sample size was estimated under the assumption of the exponential distribution for simplicity. The seed of data generation was specified as ‘123’ and 5000 trials were repeated to calculate the empirical power.

As is shown in Table 2, 591 patients are necessary to be enrolled and 423 deaths have to be observed to guarantee the target power in the two groups if the traditional single stage is considered. It is accidental that the total sample size of the two-stage design is equal to the size of the single stage design because of the fluctuation of the calculated empirical power in the simulation. But the expected number of events in the two-stage design decreases clearly. The phenomenon continues in the three-stage design, which seems better than Scenario A and B due to the more chance of early stopping for efficacy. However, in scenario C, the trial only has the probability of 2.64% of early stopping at the first stage, which is considered to be not practical and cost-effective in an actual trial. On the other hand, the chance of early stopping achieves 17.72% at the first stage and increases to 55.54% until the second stage in Scenario D, which seems more reasonable and attractive. The expected number of events is also smaller than the one of Scenario C. Although the total sample size of Scenario D is a little larger than the ones of the other 3 scenarios, it is still preferable to the sponsors considering that the trial has a larger and reasonable chance to stop earlier at the interim analyses. Therefore, the three-stage design with 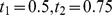 is considered as the optimal monitoring plan.

**Table 2:** The comparison of the results for different interim monitoring plans under the exponential distribution.

Scenario	Stage	*n*	*D*	*E*(*D*)	*t_i_*	*d_i_*	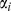	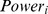	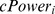
A	1	591	423	423.00	1	423	0.05	80.88%	80.88%
B	2	591	423	384.46	0.5	211	0.003051	18.18%	18.18%
					1	212	0.048999	62.68%	80.68%
C	3	594	425	359.25	1/3	141	0.000207	2.64%	2.64%
					2/3	142	0.012025	41.02%	43.66%
					1	142	0.045576	36.38%	80.04%
D	3	597	427	348.61	0.5	213	0.003047	17.72%	17.72%
					0.75	107	0.018324	37.82%	55.54%
					1	107	0.04401	25.02%	80.56%

*n*: the total sample size in two groups; *D*: the number of events to guarantee the target power in two groups; *E(D)*: the expected number of events; *t_i_*: the information time of the *i*-th interim analysis; *d_i_*: the number of events to be observed in the *i*-th stage; 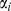: the nominal significance level of the *i*-th interim analysis; 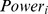: the stage-wise empirical power of the *i*-th stage; 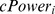: the cumulative empirical power of the *i*-th stage.

### (2) The comparison of the results for varied *γ* under the Weibull distribution

On account of the relationship among 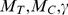 and the hazard ratio, the effects of varied shape parameters on the sample size, the number of events and other assessment indexes were explored under two scenarios: (A) varied hazard ratio due to the various *γ* with 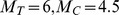; (B) varied 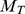 due to the various *γ* with 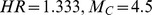. According to the last section, the three-stage design with 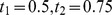 was adopted in the simulation. The seed of ‘123’ was also employed to generate the simulative data and all the indexes were calculated based on 5000 replications. The simulation results of Scenario A and B are shown in Table 3 and Table 4 respectively.

**Table 3:** The comparison of the results for varied *γ* with fixed *M_T_* and *M_C_* under the Weibull distribution (*M_T_* = 6, *M_C_* = 4.5).

*γ*	*HR*	*n*	*D*	*E*(*D*)	*t_i_*	*d_i_*	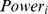	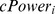
0.3	1.090	9570	4816	3955.38	0.5	2488	17.32%	17.32%
					0.75	1204	36.84%	54.16%
					1	1204	26.00%	80.16%
0.5	1.155	2967	1699	1390.45	0.5	849	18.30%	18.30%
					0.75	425	36.00%	54.30%
					1	425	26.00%	80.30%
0.55	1.171	2388	1408	1151.46	0.5	704	17.92%	17.92%
					0.75	352	37.04%	54.96%
					1	352	25.58%	80.54%
0.6	1.188	1941	1176	966.14	0.5	588	17.08%	17.08%
					0.75	294	37.22%	54.30%
					1	294	25.94%	80.24%
0.65	1.206	1626	1011	826.51	0.5	505	17.78%	17.78%
					0.75	253	37.36%	55.14%
					1	253	25.26%	80.40%
0.7	1.223	1380	879	720.34	0.5	439	17.98%	17.98%
					0.75	220	36.16%	54.14%
					1	220	25.88%	80.02%
0.75	1.241	1170	763	626.32	0.5	381	17.36%	17.36%
					0.75	191	36.84%	54.20%
					1	191	26.14%	80.34%
0.8	1.259	996	664	544.11	0.5	332	17.16%	17.16%
					0.75	166	37.90%	55.06%
					1	166	25.06%	80.12%
0.85	1.277	864	587	480.75	0.5	293	17.42%	17.42%
					0.75	147	37.44%	54.86%
					1	147	25.32%	80.18%
0.9	1.296	747	517	422.13	0.5	258	18.14%	18.14%
					0.75	129	36.84%	54.98%
					1	130	25.48%	80.46%
0.95	1.314	660	465	380.79	0.5	232	17.44%	17.44%
					0.75	116	37.24%	54.68%
					1	117	25.96%	80.64%
1	1.333	591	423	346.76	0.5	211	17.76%	17.76%
					0.75	106	36.40%	56.28%
					1	106	24.36%	80.64%
1.5	1.540	249	195	159.17	0.5	97	18.44%	18.44%
					0.75	49	36.24%	54.68%
					1	49	25.56%	80.24%
2	1.778	141	112	91.96	0.5	56	17.54%	17.54%
					0.75	28	36.50%	54.04%
					1	28	26.22%	80.26%
2.5	2.053	96	76	61.35	0.5	38	20.18%	20.18%
					0.75	19	36.74%	56.92%
					1	19	23.96%	80.88%
3	2.370	69	55	44.26	0.5	27	19.54%	19.54%
					0.75	14	37.66%	57.20%
					1	14	24.80%	82.00%
3.5	2.737	51	40	32.40	0.5	20	19.74%	19.74%
					0.75	10	36.56%	56.30%
					1	10	24.04%	80.34%
4	3.160	42	33	26.30	0.5	16	19.58%	19.58%
					0.75	8	37.48%	57.06%
					1	9	25.24%	82.30%
4.5	3.649	33	26	21.01	0.5	13	18.72%	18.72%
					0.75	6	36.48%	55.20%
					1	7	24.98%	80.18%
5	4.214	30	24	19.15	0.5	12	20.04%	20.04%
					0.75	6	40.70%	60.74%
					1	6	22.88%	83.62%
8	9.989	15	12	10.16	0.5	6	2.94%	2.94%
					0.75	3	55.56%	58.50%
					1	3	25.50%	84.00%

*γ*: the shape parameter of the Weibull distribution; *HR*: the hazard ratio; *n*: the total sample size in two groups; *D*: the number of events to guarantee the target power in two groups; *E(D)*: the expected number of events; *t_i_*: the information time of the *i*-th interim analysis; *d_i_*: the number of events to be observed in the *i*-th stage; 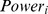: the stage-wise empirical power of the *i*-th stage; 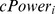: the cumulative empirical power of the *i*-th stage.

**Table 4:** The comparison of results for varied *γ* with fixed hazard ratio under the Weibull distribution (HR = 1.333).

*γ*	*M_C_/M_T_*	*n*	*D*	*E*(*D*)	*t_i_*	*d_i_*	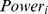	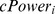
0.3	4.5/11.74	909	421	344.50	0.5	210	18.10%	18.10%
					0.75	105	36.14%	54.24%
					1	106	25.90%	80.14%
0.5	4.5/8.00	765	416	339.75	0.5	208	17.84%	17.84%
					0.75	104	37.64%	55.48%
					1	104	24.54%	80.02%
0.55	4.5/7.59	747	421	343.76	0.5	210	18.12%	18.12%
					0.75	105	36.80%	54.92%
					1	106	25.42%	80.34%
0.6	4.5/7.26	720	420	342.49	0.5	210	17.98%	17.98%
					0.75	105	37.86%	55.84%
					1	105	24.44%	80.28%
0.65	4.5/7.01	702	423	345.68	0.5	211	17.96%	17.96%
					0.75	106	37.02%	54.98%
					1	106	25.64%	80.62%
0.7	4.5/6.79	678	421	343.92	0.5	210	17.60%	17.60%
					0.75	105	37.68%	55.28%
					1	106	25.42%	80.70%
0.75	4.5/6.60	660	422	344.86	0.5	211	17.86%	17.86%
					0.75	105	37.22%	55.08%
					1	106	25.28%	80.36%
0.8	4.5/6.45	627	411	335.83	0.5	205	18.66%	18.66%
					0.75	103	35.66%	54.32%
					1	103	25.98%	80.30%
0.85	4.5/6.31	615	414	338.47	0.5	207	17.88%	17.88%
					0.75	103	37.04%	54.92%
					1	104	25.22%	80.14%
0.9	4.5/6.19	603	415	340.87	0.5	207	17.28%	17.28%
					0.75	104	36.72%	54.66%
					1	104	26.26%	80.34%
0.95	4.5/6.09	600	421	343.65	0.5	210	18.04%	18.04%
					0.75	105	37.06%	55.10%
					1	106	25.60%	80.70%
1	4.5/6.00	591	423	346.76	0.5	211	17.76%	17.76%
					0.75	106	36.40%	56.28%
					1	106	24.36%	80.64%
1.5	4.5/5.45	543	429	350.25	0.5	214	17.54%	17.54%
					0.75	107	38.00%	55.54%
					1	108	24.46%	80.00%
2	4.5/5.20	534	427	351.14	0.5	213	16.28%	16.28%
					0.75	107	38.34%	54.62%
					1	107	25.60%	80.22%
2.5	4.5/5.05	546	436	356.88	0.5	218	17.92%	17.92%
					0.75	109	36.74%	54.66%
					1	109	25.62%	80.28%
3	4.5/4.95	546	436	356.88	0.5	218	17.92%	17.92%
					0.75	109	36.74%	54.66%
					1	109	25.62%	80.28%
3.5	4.5/4.88	546	436	356.88	0.5	218	17.92%	17.92%
					0.75	109	36.74%	54.66%
					1	109	25.62%	80.28%
4	4.5/4.83	546	436	356.88	0.5	218	17.92%	17.92%
					0.75	109	36.74%	54.66%
					1	109	25.62%	80.28%
4.5	4.5/4.80	546	436	356.88	0.5	218	17.92%	17.92%
					0.75	109	36.74%	54.66%
					1	109	25.62%	80.28%
5	4.5/4.77	546	436	356.88	0.5	218	17.92%	17.92%
					0.75	109	36.74%	54.66%
					1	109	25.62%	80.28%
8	4.5/4.66	546	436	356.88	0.5	218	17.92%	17.92%
					0.75	109	36.74%	54.66%
					1	109	25.62%	80.28%

*γ*: the shape parameter of the Weibull distribution; *M_T_*: the median survival time of the treatment group; *M_C_*: the median survival time of the control group; *n*: the total sample size in two groups; *D*: the number of events to guarantee the target power in two groups; *E(D)*: the expected number of events; *t_i_*: the information time of the i-th interim analysis; *d_i_*: the number of events to be observed in the *i*-th stage; 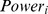: the stage-wise empirical power of the *i*-th stage; 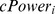: the cumulative empirical power of the *i*-th stage.

**Figure 2:**
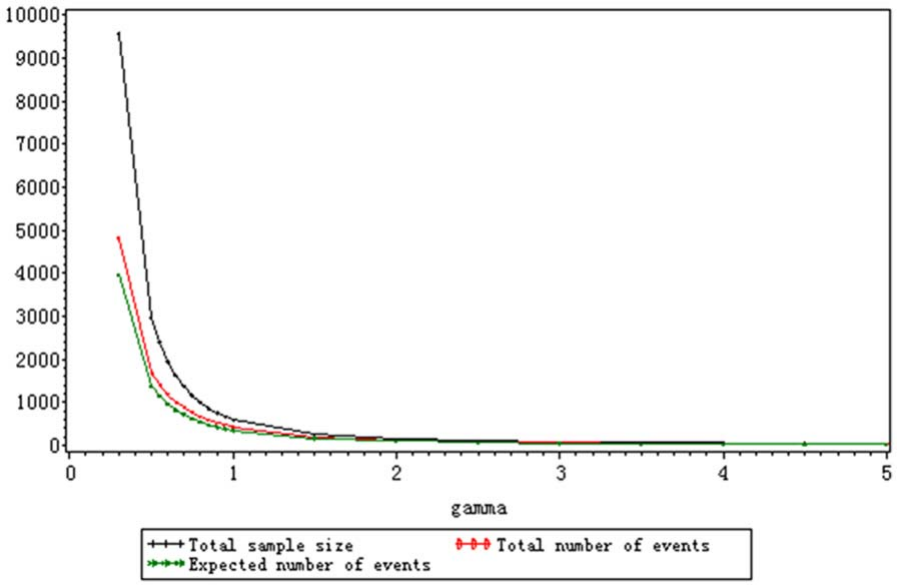
The change of *n*, *D* and *E* (*D*) for varied *γ* with fixed *M_T_* and *M_C_* under the Weibull distribution (*M_T_* = 6, *M_C_* = 4.5).

**Figure 3:**
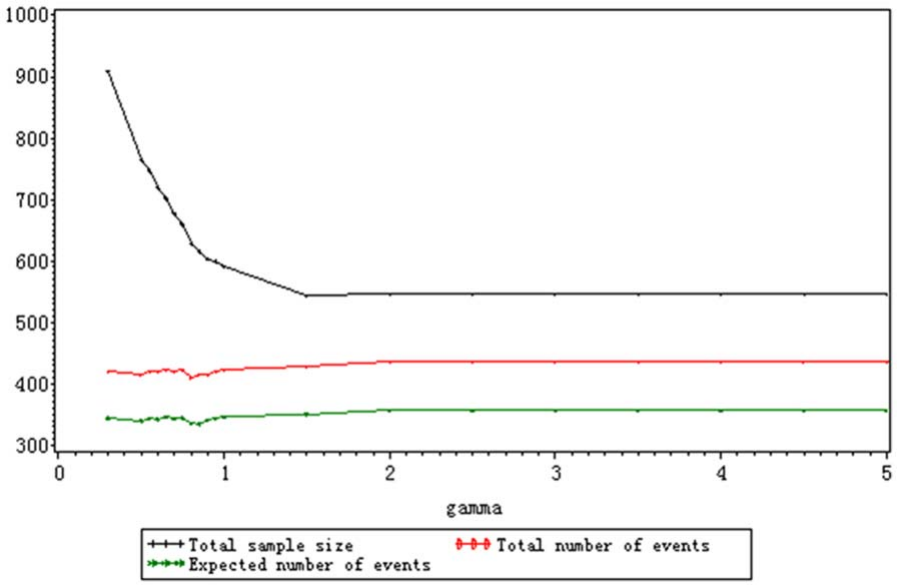
The change of *n*, *D* and *E*(*D*) for varied *γ* with fixed hazard ratio under the Weibull distribution (*HR* = 1.333).

As is shown in Table 3 and Table 4, the stage-wise empirical power and cumulative empirical power of each stage keep stable for varied shape parameters and their fluctuation are within reasonable range. The changes of shape parameters have no effect on 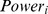 and 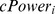 as long as the number and timing of interim analyses are fixed. The optimal interim monitoring plan under the exponential distribution also fits the trial no matter how the shape parameter varies. The total sample size, the total number and the expected number of events both decline with the increase of *γ* because the increasing *γ* leads to the rise of hazard ratio when 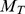 and 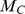 are fixed, which is depicted in Figure 2. Especially when *γ*<1, the three assessment indexes decrease dramatically. The total sample size is larger than 1000 when *γ*≤0.75 and the hazard ratio approaches to 1. The hazard ratio is so large that the total sample size is smaller than 100 when *γ*≥2.5. In Scenario B, as is shown in Figure 3, the total sample size still decrease with the rise of *γ*. But the range of total sample size is smaller than the one of Scenario A. The increasing median survival time of the treatment group, which is attributed to the decreasing *γ*, results in the larger sample size. The total number and expected number of events keep stable no matter how the shape parameter varies because the hazard ratio and target power is the same. When 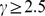, *n*, *D* and *E(D)* keeps the same and the median survival time of the treatment group approaches to the one of the control group. It is seen from the results of Scenario A and B that the total sample size is more sensitive to the hazard ratio than the median survival time of the treatment group. The total number and expected number of events only depend on the hazard ratio when the target power is a constant.

## Discussion

The sample size estimation is an important and indispensible part in trial design. It is more complicated for time-to-event data due to its ambiguous distributional form and the censored data. The different definition of information time about the time-to-event endpoint makes it more difficult to estimate the sample size. On the other hand, compared with the available theoretical methods [5]–[13], the simulation-based approach for sample size estimation is easier to implement in an actual trial and can include various practical considerations on the trial, which leads to a more accurate estimate. Therefore, a practical and simple simulation-based method is proposed to calculate the sample size of two-arm survival group sequential trial and its SAS tool is available from the Program S1. In the simulation, the inclusion of the probability of discontinuation contributes to the more accurate estimate. Both the exponential and Weibull distribution are considered to calculate the sample size. Compared with Heo's method [15], which gave a formula to calculate the sample size for single stage trial under the Weibull distribution, our method focuses on the multi-stage group sequential design. Besides the sample size, the assessment indexes, such as, 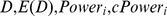, are calculated in the simulation to help determine the optimal interim monitoring plan for group sequential trial.

To implement the proposed method to calculate the sample size, one has to prespecify the survival distribution, the median survival time of the treatment and control groups, the maximum follow up time, the number of trial stages, the interim time schedule, the dropout rate, the sample size ratio, the target power, *etc*. Of them, the maximum follow up time, the dropout rate, the sample size ratio and target power are prespecified according to the suggestions of the investigators and sponsors. But the accrual information, including the accrual time, accrual rate and accrual distribution, does not have to be prespecified, which is different form the methods of some commercial softwares. That is because the accrual information depends on too many factors, e.g., the incidence rate of the disease, the number of patients in the site, the inclusion/exclusion criteria of the subject, the working efficiency of the trial monitor, *etc*. It is usually difficult for clinical investigators to predict the accrual rate and accrual distribution. If too optimistic accrual information is predicted, the estimated sample size cannot guarantee the power to detect the difference and the efficacy of the test drug is ignored. For that reason, it is presumed that the subject would enter the study only when the former one had finished the trial in the simulation from the conservative point. At the interim analysis, only the subject who finishes the trial is included for analysis and no one is still at risk in the cohort. The accrual time, accrual rate and accrual distribution do not affect the sample size and are unnecessary to define. Of course, the presumption results in a conservative and larger estimate of the sample size. But the group sequential design still makes it possible to reject the null hypothesis earlier at the interim analysis if the accrual information is better than the anticipated. In the group sequential trials, the number, timing and boundaries of interim analyses are closely related to the sample size and the number of preplanned events. Both information time and calendar time are available by employing calendar time-information time transformation. The stopping boundaries of interim analyses can be calculated conveniently by using SAS 9.2, GroupSeq Package of R, *etc* if Pocock method, O'Brien-Fleming method and the error spending method are considered. As long as the overall type I error rate is well controlled, the stopping boundaries can be determined by performing extensive simulations, too. The simulation-based method proposed here makes it possible to choose the optimal number of trial stages and interim monitoring schedule by comparing the calculated assessment indexes. Moreover, the median survival time, hazard ratio and survival distribution directly contribute to the sample size and the number of events. The median survival time and hazard ratio of the two groups reflect the efficacy of the test drug and satisfy 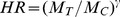 when the Weibull distribution is assumed. From the simulation results in Table 3 and 4, it is seen that the number of events, which should be observed to guarantee the target power, only depends on the hazard ratio. The number of subjects to be accrued is more sensitive to the hazard ratio than the median survival time. When HR = 1.333 and 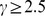, the sample size keeps the same no matter how the shape parameter varies. But the test drug cannot length the survival time of the patient significantly compared to the placebo. However, when 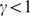, the patients may benefit from the prolonged survival time though a larger sample size is needed. On the other hand, when the survival time of the two groups is fixed, the hazard ratio, which varies with the shape parameter of the Weibull distribution, directly results in the sample size. When 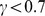, too low hazard ratio leads to so large sample size and it may be unnecessary to start the trial due to the poor efficacy. When 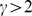, too small sample size because of the high hazard ratio makes it possible to shorten the follow up time and accrue more patients. The whole study process of the drug will be quickened and the trial costs may be saved.

In an actual clinical trial, the median survival time is an intuitive endpoint to the clinical investigators and reflects the benefits of the patient from the drug. It is relatively easy for the clinician to estimate the median survival time of the treatment and control groups based on the literature review and the pre-studies on the drug. The hazard ratio is referred to as the hazard rate of the event of the control group to the treatment group. For a clinical trial with the hazard ratio of *HR*, it is thought that the test drug is able to decrease (1-1/*HR*) risk of the event compared to the control group. It is also possible for the clinical investigator to estimate the hazard ratio with the help of the biostatistician and other participants according to the pre-studies and the expectations on the test drug. Therefore, when the Weibull distribution, which is more appropriate to describe the time-to-event endpoint for the included shape parameter, is assumed in a trial, it is suggested that the acceptable range of the median survival time of the test drug and the hazard ratio should be estimated at first in order to calculate 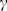. Let 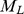 be the minimum significant median survival time of the test drug from the clinical perspective and 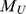 be the largest expected median survival time. The estimated range of the hazard ratio is 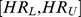, where 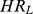 and 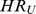 denote the lower and upper limit of the estimated hazard ratio. According to the simulation results, the shape parameter of the Weibull distribution is calculated by
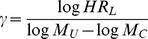
(7)from the conservative point, where 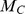 denotes the median survival time of the control group. We still take the metastatic colorectal cancer clinical trials we have mentioned above as an example to illustrate how to estimate 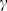 explicitly. It was predicted that the test drug could lengthen the median OS by 2.5 months at best and the median OS of the placebo group was 4.5 months. The test drug was expected to decrease 25%-30% risk of death compared to the placebo. Accordingly, the range of the hazard ratio was [1.333, 1.429]. The shape parameter of the Weibull distribution was estimated as 0.651 by using Eq.(7). When the three-stage group sequential design with 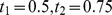 was employed and O'Brien-Fleming boundaries were considered, 696 subjects and 420 deaths were necessary to achieve 80% power to detect the difference.

Whether the fluctuation of the sample sizes for various random seeds leads to the inaccurate estimate is another vital concern about the simulation-based approach. To explore the problem, 10 different random seeds were specified respectively to calculate the sample sizes under the assumption of the exponential distribution for the cancer clinical trial we have introduced in the example. The three-stage design with 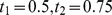 was considered to calculate the sample size. The derived sample sizes range from 588 to 606. The mean and standard deviation of them are 598.2 and 5.13. Correspondingly, the number of deaths ranges from 421 to 434 with the mean of 428.1 and standard deviation of 3.81. It seems that 10 simulations are enough to provide a robust estimate. Therefore, in order to get the robust estimate of the sample size, 10+ simulations with various seeds are suggested and their mean is recommended as the final sample size. Moreover, we find that the sample sizes calculated under the exponential distribution and the Weibull distribution with the shape parameter of 1 are not equal exactly even if the same seed was specified, which is seen in Table 2 and 3. It is because different SAS functions were called to generate the streams of random number, which results in the differences of derived sample sizes. But the mean sample size under the Weibull distribution with 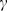 of 1, which was estimated from 10 simulations with the same seeds as the simulations of the exponential distribution, is 601.8. It is very close to the mean sample size under the exponential distribution.

However, adaptive design, which is considered to be an extension of group sequential design, has been increasingly applied in the recent years. It allows sample size re-estimation after interim analysis to cope with the inaccurate estimate on the efficacy of the drug when designing the trial. The simulation-based approach introduced in this article, which was initiated from the group sequential design, can also be applied in adaptive design by employing the following strategy. In the planning phase of the trial, a series of possible scenarios about the efficacy of the test drug are assumed in advance and the sample size scheme for each possible scenario is calculated by employing %n_gssur. After the interim analysis, the investigator is able to choose the suitable sample size from the scheme for the subsequent stage according to the results of interim analysis. In the strategy, no modification of the program is necessary. The strategy protects the integrity of adaptive design to a greater extent than the methods of sample size re-calculation after interim analysis. But the error-spending boundaries may be not fit in the strategy and they have to be determined by extensive simulations. The detailed procedures for stopping boundary determination and sample size scheme calculation can be seen in [27]. On the other hand, the minor modifications of the program are inevitable if the sample size re-calculation after interim analysis is considered. To run the macro for sample size re-calculation, the interim data is necessary and the statistical test procedure has to be modified. Here, the log-rank test is still suggested for interim/final analysis and the derived *p*-values are pooled by employing the inverse normal method [26], [28]. Depending on these modifications on the SAS program, it can be further applied to re-estimate the sample size in adaptive design.

## Supporting Information

Program S1 **The detailed codes and sample call of the SAS program %n_gssur.**
(SAS)
